# Postoperative Complications After Indocyanine Green-Guided Colorectal Cancer Surgery: Report of an Unexpected Severity Signal From a Prospective Cohort Study With Cluster-Based Allocation

**DOI:** 10.7759/cureus.110974

**Published:** 2026-06-16

**Authors:** Mihaela C Misca, Eduard Catrina, Sorin Aldoescu, Mihaela Vilcu, Dana R Boanta, Iulian Brezean

**Affiliations:** 1 General Surgery, Carol Davila University of Medicine and Pharmacy, Bucharest, ROU; 2 General Surgery, Spitalul Clinic Dr. C. I. Parhon, Bucharest, ROU

**Keywords:** anastomotic leak, clavien-dindo classification, cluster allocation, colorectal cancer, fluorescence angiography, indocyanine green, postoperative complications, right colectomy

## Abstract

Background and objectives: Indocyanine green fluorescence angiography (ICG-FA) is increasingly used in colorectal cancer surgery to assess intraoperative anastomotic perfusion and potentially reduce postoperative complications, particularly anastomotic leakage. However, the magnitude and consistency of the benefit remain debated, especially regarding complication severity. We report the impact of ICG-FA on postoperative complications in a single-center prospective comparative cohort study with cluster-based group allocation, with particular focus on an unexpected severity signal observed in our data.

Methods: Between January 2020 and December 2025, 315 patients undergoing elective resection for colorectal cancer were prospectively enrolled at a tertiary surgical department in Bucharest, Romania. Group allocation was determined by the temporal availability of the operating theater equipped with a near-infrared laparoscopic tower (one to two days per week, dedicated to extensive laparoscopic procedures). Sixty-six patients underwent ICG-FA-guided resection (3 mg intravenous bolus, standard institutional protocol); 247 underwent conventional resection. Primary outcomes were overall postoperative complications (Clavien-Dindo grade II or higher) and severe complications (anastomotic leak or Clavien-Dindo grade III or higher). Statistical analyses were performed with JASP version 0.94.5.

Results: The overall complication rate was 31.8% in the ICG group versus 39.4% in the non-ICG group, a numerical reduction that did not reach statistical significance (chi-square = 1.26, p = 0.26). The postoperative hospital stay was significantly shorter in the ICG group (6.92 ± 4.78 days vs. 9.21 ± 8.07 days, p < 0.01). Among patients who developed any postoperative complication (n = 119), the proportion of severe complications was significantly higher in the ICG arm: 28.57% (6/21) versus 9.18% (9/98) in the non-ICG arm (chi-square = 5.90, p = 0.02; Fisher log odds ratio = 1.38, p = 0.03). Multivariate logistic regression identified right-colon tumor location as the strongest independent predictor of postoperative complications (odds ratio = 7.01, p < 0.01), with preoperative albumin showing a borderline protective effect (odds ratio = 1.94, p = 0.05).

Conclusions: In our cohort, ICG-FA was associated with shorter hospital stay and a non-significant reduction in overall complications, but with an unexpected and statistically significant increase in the proportion of severe complications among patients who developed any complication. We discuss this counter-intuitive finding openly, considering three plausible mechanisms: statistical fragility related to small event numbers, confounding by procedural complexity inherent to the cluster-based design, and a real component possibly related to false reassurance and learning curve effects. Honest reporting of such signals is essential for refining the appropriate clinical use of ICG-FA. Larger, stratified, prospective studies are needed to clarify whether the observed signal reflects a true phenomenon or chance.

## Introduction

Anastomotic leakage remains the most feared complication of colorectal cancer surgery, with reported incidence between 3% and 20% depending on tumor location and surgical technique, and with significant consequences in terms of mortality, length of hospital stay, oncologic outcomes, and quality of life [1,2]. Among the technologies that have emerged in the past decade with the aim of reducing this risk, indocyanine green fluorescence angiography (ICG-FA) has gained particular interest because it provides real-time visual assessment of perfusion at the anastomotic line, with the potential to inform intraoperative decisions such as extending the proximal resection margin, modifying the line of transection, or converting a planned anastomosis to a temporary or permanent stoma [3-5].

A growing body of evidence supports the use of ICG-FA in colorectal surgery. Randomized controlled trials such as PILLAR III [6], the FLAG trial [7], and the EssentiAL trial [8], along with large propensity score-matched cohorts by Watanabe [9], Hasegawa [10], and Foo [11], have suggested reductions in anastomotic leak rates, particularly in low rectal resections. Meta-analyses pooling data from thousands of patients have reported pooled relative risks for anastomotic leak around 0.45, representing approximately a 50% relative risk reduction [12,13]. Other studies, however, have failed to demonstrate a statistically significant benefit in the overall cohort and have noted that the effect of ICG-FA may be modulated by tumor location, surgeon experience, learning curve, and the absence of standardized protocols [14,15].

Beyond anastomotic leakage, ICG-FA may influence the broader spectrum of postoperative complications, including overall morbidity, rate of reintervention, and complication severity as graded by the Clavien-Dindo classification. The literature on these broader outcomes is more heterogeneous. While some studies report a global reduction in postoperative morbidity associated with ICG use [12,16], others report no significant differences [15], and few have specifically addressed whether ICG influences the distribution of complication severity rather than just overall incidence.

The aim of the present study was to report a single tertiary center experience with ICG-FA in colorectal cancer surgery, with a focus on postoperative complications. We pay particular attention to a counterintuitive observation that emerged from our analysis: although the overall complication rate was numerically lower in the ICG group, the proportion of severe complications among patients who developed any complication was significantly higher in the ICG arm. We report this finding transparently and discuss three plausible mechanisms in the belief that honest reporting of such signals is essential to a balanced understanding of the clinical role of ICG-FA.

## Materials and methods

Study design and setting

We conducted a single-center prospective comparative cohort study with cluster-based group allocation at the Department of Surgery, Clinical Hospital "Dr. I. Cantacuzino," Bucharest, Romania, between January 2020 and December 2025. The study was approved by the institutional ethics committee and conducted in accordance with the Declaration of Helsinki. All patients provided written informed consent for the procedure and for the use of their data for research purposes.

Group allocation

During the study period, the department operated with a single laparoscopic tower equipped with an integrated near-infrared (NIR) fluorescence detection system, available for one to two days per week according to a pre-established institutional schedule. On these days, the dedicated operating theater was reserved for extensive laparoscopic procedures (estimated duration over three hours). Patients scheduled on these days were assigned to the ICG-FA arm; patients scheduled on the remaining days were assigned to the conventional surgery arm. Allocation was therefore determined exclusively by theater availability, not by surgeon preference or intraoperative assessment of case complexity. We acknowledge that this cluster-based allocation generates an inherent imbalance between groups regarding the proportion of laparoscopic versus open procedures, which we address explicitly in our analyses and discussion.

Patient population

During the study period, 420 patients with colorectal pathology were evaluated at the department. After application of the inclusion and exclusion criteria detailed in Table 1, 315 patients were included in the final analysis: 66 of 315 (21.0%) in the ICG-FA group and 249 of 315 (79.0%) in the conventional surgery group.

**Table 1:** Inclusion and exclusion criteria.

Category	Criterion
Inclusion criteria	Histopathologically confirmed adenocarcinoma of the colon or rectum (preoperative colonoscopic biopsy).
	Indication for elective resection of the primary tumor as established by the institutional multidisciplinary tumor board.
	Age over 18 years.
	Signed written informed consent for the procedure and for the use of data for research purposes.
	Absence of known contraindications to ICG administration (iodine allergy, severe thyroid dysfunction, severe hepatic insufficiency).
Exclusion criteria	Non-oncologic colorectal pathology (inflammatory bowel disease without concomitant cancer, complicated diverticulitis, uncomplicated polyposis).
	Surgical intervention contraindicated due to severe medical comorbidities (ASA IV-V) or unresectable metastatic disease managed with systemic oncologic therapy alone.
	Palliative procedures only, without resection of the primary tumor (e.g., diverting stoma without resection).
	Refusal to participate in the study or subsequent withdrawal of consent.

ICG administration protocol

In the ICG-FA arm, indocyanine green was administered as a fixed-dose intravenous bolus of 3 mg, reconstituted in sterile water for injection. This dose is at the lower end of the range reported in the international literature (typically 2.5 to 10 mg per administration) and was chosen based on institutional experience showing adequate fluorescence signal and a favorable safety profile. The first administration was performed immediately before proximal transection of the colon to confirm adequate perfusion at the line of transection. A second administration was performed immediately after anastomosis construction to confirm perfusion at the anastomotic line. Decisions regarding modification of the surgical plan (extension of the resection margin, conversion from anastomosis to stoma) were made by the operating surgeon, integrating the fluorescence information with the clinical context.

Variables and outcome definitions

For each patient, we prospectively recorded demographic variables (age, sex), oncologic variables (tumor location, clinical TNM stage, and neoadjuvant treatment), preoperative biological parameters (hemoglobin, leukocytes, C-reactive protein, albumin, carcinoembryonic antigen, and CA 19-9), operative variables (type of approach and type of procedure), and postoperative outcomes (total hospital stay, postoperative hospital stay, postoperative complications classified per Clavien-Dindo, and occurrence of anastomotic leak as defined by the International Study Group of Rectal Cancer [1]).

The primary outcome of the present analysis was the rate of overall postoperative complications, defined as any complication graded Clavien-Dindo grade II or higher within 30 days of surgery. The secondary outcome was the rate of severe complications, defined as the occurrence of an anastomotic leak or Clavien-Dindo grade III or higher, analyzed both for the entire cohort and as the proportion of severe complications among patients who developed any complication.

Statistical analysis

All statistical analyses were performed with JASP Statistics, version 0.94.5 (University of Amsterdam, The Netherlands). Continuous variables were described as mean ± standard deviation, and categorical variables as absolute frequencies and percentages. The Shapiro-Wilk test was used to assess normality. Continuous variables were compared using the Student t-test or the Mann-Whitney U test, depending on distribution; effect sizes were quantified using Cohen’s d for the t-test and rank biserial correlation for the Mann-Whitney test. Categorical variables were compared using the chi-square test of independence, with degrees of freedom and sample size reported as chi-square (df, N); effect sizes were quantified using the Phi coefficient (two-by-two tables) and Cramer V (larger tables). The Fisher's exact test was substituted when expected frequencies were below five. Binary logistic regression models were constructed to identify independent predictors of (a) ICG use and (b) postoperative complications, with the inclusion of relevant clinical variables. Model adequacy was assessed by Nagelkerke R-squared, deviance, AIC, and BIC; predictive performance was assessed by accuracy, area under the receiver operating characteristic curve (AUC), sensitivity, and specificity. Statistical significance was set at p < 0.05.

## Results

Baseline characteristics of the cohort

The cohort comprised 315 patients (mean age 66.27 ± 11.10 years), 145 of 315 women (46.0%), and 170 of 315 men (54.0%). Tumor location distribution was as follows: 31 of 315 right colon (9.8%), 12 of 315 transverse colon (3.8%), 68 of 315 left colon (21.6%), and 204 of 315 rectum (64.7%). Of the 66 patients in the ICG group, the mean age was 63.56 ± 13.25 years; of the 249 in the non-ICG group, 66.98 ± 10.28 years (analysis of variance p = 0.05; post-hoc Tukey test not significant). Patients in the ICG group had significantly higher mean preoperative hemoglobin (13.15 ± 2.01 g/dL vs. 11.98 ± 2.22 g/dL; Mann-Whitney p < 0.01, Cohen d = 0.30), significantly lower leukocyte counts (7.19 ± 2.67 vs. 8.18 ± 3.38 times 10³ per microliter; p < 0.05, d = 0.17), and significantly lower preoperative carcinoembryonic antigen (3.69 ± 6.82 vs. 14.49 ± 46.68 ng/mL; p < 0.01, d = 0.32). Preoperative C-reactive protein and CA 19-9 did not differ significantly (p = 0.28 and p = 0.68, respectively). Distribution of clinical TNM stages showed a tendency toward more advanced stages in the non-ICG group: 29 of 30 stage IV patients (96.67%) belonged to the non-ICG group. Neoadjuvant treatment was administered in similar proportions (p = 0.71). Tumor location and patient sex were not associated with ICG use (chi-square (3, N = 315) = 3.36, p = 0.34, Cramer's V = 0.10; and chi-square (1, N = 315) = 0.52, p = 0.47, phi = 0.04, respectively). Full baseline characteristics are presented in Table 2.

**Table 2:** Baseline clinico-demographic characteristics of the cohort, by group. ICG: indocyanine green; SD: standard deviation; CRP: C-reactive protein; CEA: carcinoembryonic antigen; CA 19-9: carbohydrate antigen 19-9; TNM: tumor-node-metastasis.

Variable	ICG group (n = 66)	Non-ICG group (n = 249)	Test, p-value, effect size
Age (years, mean ± SD)	63.56 ± 13.25	66.98 ± 10.28	ANOVA, p = 0.05 (borderline); Tukey post-hoc not significant
Sex, n (%)			χ²(1, N = 315) = 0.52, p = 0.47, Phi = 0.04
Female	33 of 66 (50.0%)	112 of 249 (45.0%)	
Male	33 of 66 (50.0%)	137 of 249 (55.0%)	
Tumor location, n (%)			χ²(3, N = 315) = 3.36, p = 0.34, Cramer V = 0.10
Left colon	14 of 66 (21.2%)	54 of 249 (21.7%)	
Transverse colon	0 of 66 (0.0%)	12 of 249 (4.8%)	
Right colon	7 of 66 (10.6%)	24 of 249 (9.6%)	
Rectum	45 of 66 (68.2%)	159 of 249 (63.9%)	
of which lower rectum	8 of 45 (17.8%)	31 of 159 (19.5%)	χ², p = 0.80 (not significant)
Total hospital stay (days, mean ± SD)	9.77 ± 4.69	12.01 ± 7.73	Mann-Whitney U, p < 0.01
Postoperative hospital stay (days, mean ± SD)	6.92 ± 4.78	9.21 ± 8.07	Mann-Whitney U, p < 0.01
Preoperative hemoglobin (g/dL, mean ± SD)	13.15 ± 2.01	11.98 ± 2.22	Mann-Whitney U, p < 0.01, Cohen d = 0.30
Preoperative CRP (mg/L, mean ± SD)	32.93 ± 55.74	36.04 ± 52.59	Mann-Whitney U, p = 0.28, Cohen d = 0.09
Preoperative leukocytes (×10³/µL, mean ± SD)	7.19 ± 2.67	8.18 ± 3.38	Mann-Whitney U, p < 0.05, Cohen d = 0.17
Preoperative CEA (ng/mL, mean ± SD)	3.69 ± 6.82	14.49 ± 46.68	Mann-Whitney U, p < 0.01, Cohen d = 0.32
Preoperative CA 19-9 (U/mL, mean ± SD)	17.79 ± 23.82	22.30 ± 47.03	Mann-Whitney U, p = 0.68 (not significant)
Clinical TNM stage, n (%)			Insufficient cell counts for χ² (descriptive only)
Stage 0/I	18 of 66 (27.3%)	27 of 249 (10.8%)	
Stage II/III	4 of 66 (6.1%)	12 of 249 (4.8%)	
Stage IV	1 of 66 (1.5%)	29 of 249 (11.6%)	
Other / unspecified	0 of 66 (0.0%)	3 of 249 (1.2%)	
Neoadjuvant treatment, n (%)			χ²(1, N = 315) = 0.14, p = 0.71
Yes	22 of 66 (33.3%)	77 of 249 (30.9%)	
No	44 of 66 (66.7%)	172 of 249 (69.1%)	

Length of hospital stay

Total hospital stay was significantly shorter in the ICG group (9.77 ± 4.69 days) than in the non-ICG group (12.01 ± 7.73 days; Mann-Whitney p < 0.01). Postoperative hospital stay showed the same pattern: 6.92 ± 4.78 days versus 9.21 ± 8.07 days (Mann-Whitney p < 0.01). The absolute difference of approximately 2.3 days in postoperative stay corresponds to a relative reduction of approximately 25%.

Distribution of operative approach

Of the 315 patients, 217 of 315 (68.9%) underwent open surgery, and 98 of 315 (31.1%) underwent laparoscopic surgery. The cluster-based allocation, combined with the institutional protocol of reserving NIR theater days for extensive laparoscopic procedures, generated a strong association between ICG use and laparoscopic approach: 41 of 98 laparoscopic procedures (41.84%) benefited from ICG, compared with only 25 of 217 open procedures (11.52%) (chi-square (1, N = 315) = 37.46; p < 0.001; Cramer V = 0.34). Of the 66 patients who received ICG, 41 of 66 (62.1%) underwent laparoscopic surgery, while laparoscopy represented only 98 of 315 (31.1%) of the overall cohort. This procedural imbalance is the principal confounder addressed in the discussion.

Overall postoperative complications

Overall, postoperative complications occurred in 119 of 315 patients (37.8%). The complication rate was numerically lower in the ICG group (21 of 66 patients, 31.8%) than in the non-ICG group (98 of 249 patients, 39.4%), but the difference did not reach statistical significance (chi-square (1, N = 315) = 1.26; p = 0.26; Cramer V = 0.06). Notably, 45 of 66 patients in the ICG group (68.18%) developed no complication, compared with 151 of 249 patients in the non-ICG group (60.64%), and multiple complications (three or more per patient) were less frequent in the ICG group (4 of 66, 6.07%, vs. 19 of 249, 7.63%). The full distribution of the number of complications per patient is presented in Table 3.

**Table 3:** Distribution of the number of postoperative complications per patient, by group. Complications are defined as Clavien-Dindo grade II or higher within 30 days of surgery. ICG: indocyanine green.

Number of complications	ICG group, n (%) of 66	Non-ICG group, n (%) of 249	Total, n (%) of 315
None	45 (68.18%)	151 (60.64%)	196 (62.22%)
1	11 (16.67%)	48 (19.28%)	59 (18.73%)
2	6 (9.09%)	31 (12.45%)	37 (11.75%)
3	3 (4.55%)	6 (2.41%)	9 (2.86%)
4	1 (1.52%)	10 (4.02%)	11 (3.49%)
5	0 (0.00%)	2 (0.80%)	2 (0.63%)
6	0 (0.00%)	1 (0.40%)	1 (0.32%)

Severe complications: the central finding

Among the 119 patients who developed any postoperative complication, we analyzed the proportion of severe complications (anastomotic leak or Clavien-Dindo grade III or higher). Of the 21 patients with complications in the ICG group, 6 (28.57%) had severe complications; of the 98 patients with complications in the non-ICG group, 9 (9.18%) had severe complications (chi-square (1, N = 119) = 5.90; p = 0.02; Cramer V = 0.22). The Fisher exact test confirmed the significance of the association (log odds ratio = 1.38; 95% confidence interval -0.21 to 2.54; p = 0.03). Reported on the whole cohort, severe complications occurred in 6 of 66 patients (9.1%) in the ICG group versus 9 of 249 patients (3.6%) in the non-ICG group, a numerical difference that remains substantial despite the small absolute numbers.

**Figure 1:**
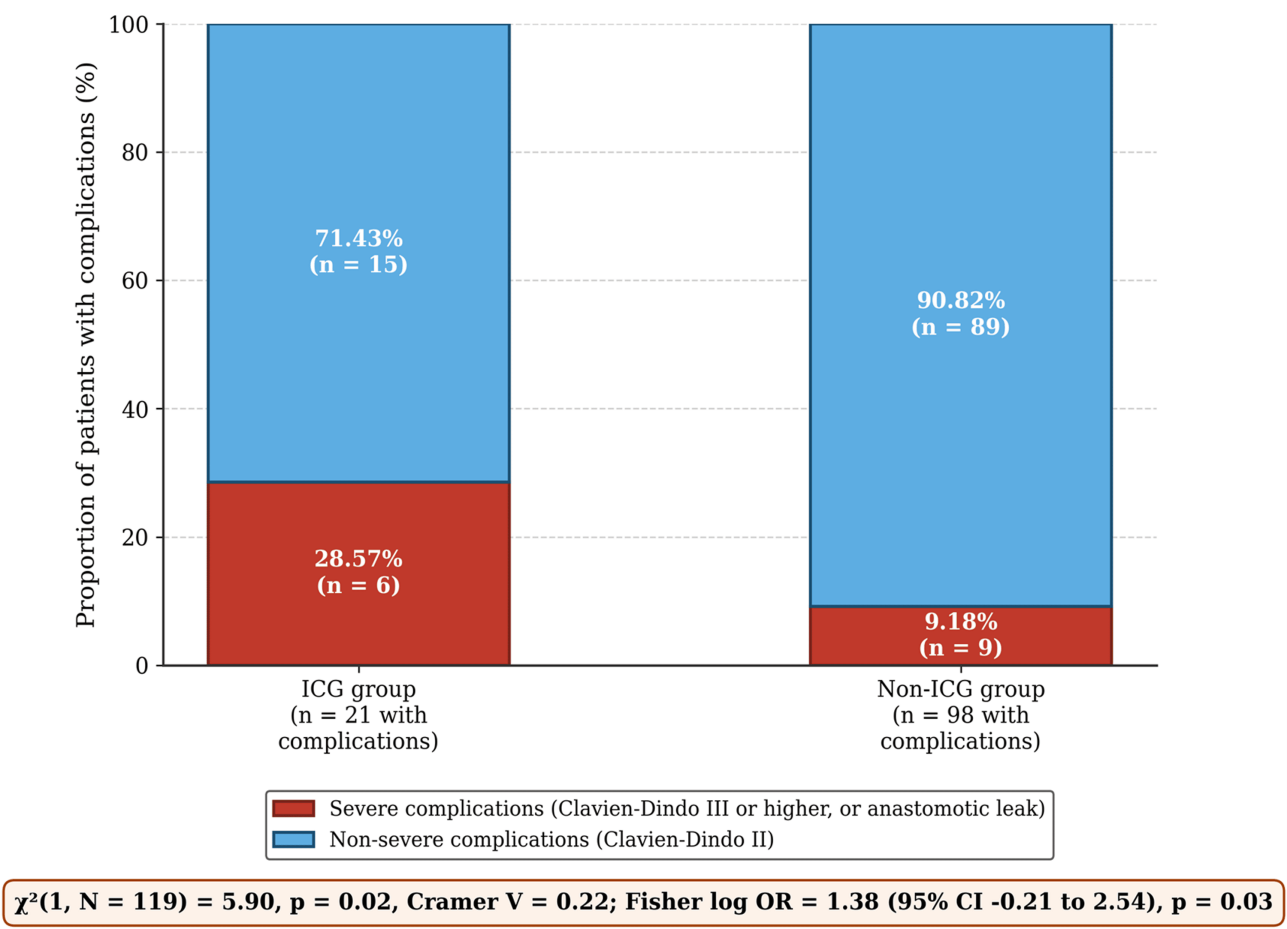
Distribution of severe versus non-severe complications between the ICG-FA and non-ICG groups, restricted to patients who developed any postoperative complication (n = 119). The chart depicts proportions and absolute frequencies. Statistical annotations: chi-square test of independence with degrees of freedom, sample size, p-value, and Cramer's V effect size; Fisher's exact test log odds ratio with 95% confidence interval and p-value.

The distribution of severe versus non-severe complications between groups is shown in Figure 1.

Predictors of postoperative complications (multivariate analysis)

A binary logistic regression model with age, preoperative albumin, preoperative CRP, and tumor location as predictors of any postoperative complication was statistically significant overall (delta chi-square [6] = 17.51; p < 0.001), although with modest explanatory power (Nagelkerke R-squared = 0.09). The strongest independent predictor was tumor location in the right colon (odds ratio = 7.01; p < 0.01). Preoperative albumin showed a borderline protective effect (odds ratio = 1.94 per g/dL increase; p = 0.05). Age (odds ratio = 1.00; p = 0.95), preoperative CRP (odds ratio = 1.00; p = 0.78), and tumor location in the transverse colon (odds ratio = 3.06; p = 0.15) or rectum (odds ratio = 1.03; p = 0.94) were not independent predictors.

## Discussion

This single-center prospective comparative cohort study with cluster-based group allocation evaluated the effect of intraoperative ICG-FA on postoperative complications in colorectal cancer surgery. Three findings emerged: a significant reduction in postoperative hospital stay associated with ICG use, a non-significant trend toward fewer overall complications in the ICG arm, and an unexpected statistically significant increase in the proportion of severe complications among patients who developed any complication in the ICG arm. We discuss each finding, in turn, with particular attention to the third, which we believe deserves transparent reporting and careful mechanistic analysis.

The reduction in postoperative hospital stay (6.92 vs. 9.21 days; p < 0.01) is consistent with pooled meta-analytical estimates reporting mean differences in favor of the ICG group [13]. The magnitude in our cohort is larger than typical pooled estimates but consistent with individual large studies on rectal resections [9,16]. This effect cannot be attributed exclusively to ICG, however, because our cluster-based allocation systematically over-represents laparoscopic procedures in the ICG arm, and minimally invasive surgery is independently associated with shorter hospital stays. The contribution of the laparoscopic approach to the observed reduction is, therefore, likely substantial.

The non-significant trend toward fewer overall complications in the ICG arm (31.8% vs. 39.4%) is consistent with the heterogeneous literature on this outcome. Most meta-analyses report a modest but statistically significant reduction in overall morbidity associated with ICG use [12,13], but the magnitude varies widely across studies, and individual randomized trials have failed to demonstrate significance in the entire cohort [15]. Our results are compatible with a true modest benefit that our study is underpowered to detect: with 66 patients in the ICG arm, detecting an absolute reduction of eight percentage points in overall complications would require several hundred patients per arm.

The central, counterintuitive finding of our study is the significantly higher proportion of severe complications among patients who developed any complication in the ICG arm (28.57% vs. 9.18%; p = 0.02). We have considered several explanations and present them transparently because we believe that an honest mechanistic analysis of unexpected findings is more valuable to the literature than dismissal as an artifact.

First, the result may be partially statistical. The absolute number of events is small (n = 15 severe complications, of which 6 are in the ICG arm), and the Fisher 95% confidence interval for the log odds ratio (-0.21 to 2.54) crosses zero at the lower bound, indicating statistical significance that is real but fragile. The analysis is also conditional on the presence of postoperative complications, which introduces a form of collider bias [17]: conditioning on a variable influenced by both factors of interest (ICG use and complication severity) can generate spurious associations.

Second, the result may reflect confounding by procedural complexity. Our cluster-based allocation systematically channeled ICG into extensive laparoscopic procedures, which in our cohort included a high proportion of low rectal resections. Low rectal resections are intrinsically associated with a higher risk of anastomotic leak and, when a leak occurs, with a higher severity grade due to the technical difficulty of reintervention, proximity to the sphincter complex, and consequences of pelvic sepsis [7,9,10]. In this view, the higher severity of complications in the ICG arm is a marker of case complexity, not of ICG-induced harm.

Third, and this is the explanation we want to address with particular candor, the result may reflect a real, clinically meaningful phenomenon: false reassurance induced by the fluorescence image. When the surgeon visualizes apparently satisfactory perfusion at the transection line, it is plausible that, in some cases, this visual confirmation attenuates the impulse to add protective measures: defunctioning ileostomy, additional mobilization of the splenic flexure, or conversion to a Hartmann procedure. In other words, ICG may, in selected situations, encourage the construction of an anastomosis that otherwise would not have been attempted. When such an anastomosis fails, the resulting complication is, by its nature, more severe. This hypothesis is compatible with the observation by Mizrahi and colleagues, who reported a change in surgical plan in 13.3% of low anterior resections based on fluorescence imaging [18], and with the FLAG trial subanalysis, which demonstrated ICG benefit concentrated on low anastomoses [7], the very setting in which, without objective perfusion assessment, the surgeon may have chosen a protective stoma.

We also considered the contribution of the learning curve. Our institutional adoption of ICG-FA followed the classical pattern of technology uptake: 7.25% of cases in 2021, rising to 32.73% in 2023, and stabilizing around 30% thereafter. In the early phase of adoption, fluorescence image interpretation is necessarily more variable, and intraoperative decisions based on it may be less calibrated. McEntee and colleagues have recently emphasized that the subjectivity of qualitative ICG assessment is a major source of inter-observer variability and a recognized limitation of the literature on this technology [19]. The use of a fixed 3 mg dose, the absence of quantitative parameters such as maximum fluorescence intensity, time to onset of fluorescence, and time to half-maximum [20,21], and the absence of a standardized institutional protocol for plan modification based on the fluorescence image all contribute to this calibration challenge.

We, therefore, propose that the observed severity signal is most likely a combination of these three factors: statistical fragility, confounding by procedural complexity, and a real component related to false reassurance and learning curve. None of these factors, taken alone or together, justify dismissing the signal as an artifact, nor do they justify extrapolating it as evidence against ICG. The most honest interpretation, in our view, is that our data identify a question that deserves further investigation: does ICG-FA, in some clinical contexts, encourage the construction of anastomoses with marginal indications, and if so, what are the criteria that should guide its use?

The strongest independent predictor of postoperative complications in our cohort was right-colon tumor location (odds ratio = 7.01; p < 0.01), an observation that, although secondary to our main analysis, deserves comment. The literature has increasingly recognized that ileocolic anastomoses after right hemicolectomy, traditionally considered safer than colorectal anastomoses, can present a significant rate of leak and septic complications, especially in elderly patients with comorbidities, malnutrition, or emergency presentation. The European audit by the European Society of Coloproctology on 3,208 right hemicolectomies reported an anastomotic complication rate of 8.1% [22]. Our data add to the growing recognition that right-colon resections are a population that deserves more attention from the perspective of ICG-FA. To date, the use of ICG in right colectomy has been limited [15], and the question of whether this technology could provide meaningful benefit specifically in this subpopulation remains open and worth investigating.

Limitations

Our study has several limitations that deserve explicit acknowledgment. First, the cluster-based allocation, although superior to retrospective observational design, generates an inherent imbalance between groups regarding operative approach, which confounds isolation of the specific ICG effect. Second, the size of the ICG group (n = 66) is modest compared with major international studies, limiting statistical power for detecting modest but clinically meaningful differences. Third, we did not use quantitative fluorescence parameters or a standardized intraoperative decision protocol, limitations consistent with the general state of the literature [19]. Fourth, the data do not include detailed information on operative duration, laparoscopic-to-open conversion, or application of enhanced recovery protocols, factors that may have modulated observed results.

Despite these limitations, the study has two strengths that justify its publication. First, the prospective collection of data within a cluster-based allocation design represents a methodological step toward true randomization in a real-world institutional setting. Second, the transparent reporting of an unexpected severity signal, with mechanism-oriented discussion, aims to contribute to a more nuanced understanding of ICG-FA in colorectal surgery, rather than to either confirm or refute the consensus of the global literature.

## Conclusions

In a single-center prospective comparative cohort with cluster-based group allocation, ICG fluorescence angiography during colorectal cancer surgery was associated with a significantly shorter postoperative hospital stay and a non-significant trend toward fewer overall postoperative complications. Unexpectedly, the proportion of severe complications among patients who developed any complication was significantly higher in the ICG arm. We analyzed this counterintuitive finding by considering three plausible mechanisms: statistical fragility, confounding by procedural complexity, and a real component related to false reassurance and learning curve. We argue that transparent reporting of such signals is essential to refine the clinical use of ICG-FA in colorectal surgery. We do not interpret our results as evidence against ICG but as a call for further studies, larger and stratified by tumor location, with standardized administration protocols and quantitative analysis of fluorescence curves that can either confirm or refute the signal observed in our cohort.
